# Bioinformatic analysis reveals an exosomal miRNA-mRNA network in colorectal cancer

**DOI:** 10.1186/s12920-021-00905-2

**Published:** 2021-02-27

**Authors:** Jun Ma, Peilong Wang, Lei Huang, Jianxia Qiao, Jianhong Li

**Affiliations:** 1 Department of Thoracic Surgery, Heji Hospital Affiliated To Changzhi Medical College, Changzhi, 046011 Shanxi China; 2 Department of Endoscopy, Heji Hospital Affiliated To Changzhi Medical College, Changzhi, 046011 Shanxi China; 3 Department of Pathology, Heping Hospital Affiliated To Changzhi Medical College, 160 East Jiefang Street, Changzhi, 046000 Shanxi China

**Keywords:** Colorectal cancer, Exosome, Differential expression analysis, Machine learning, MiRNA

## Abstract

**Background:**

Exosomes play important roles in angiogenesis, drug resistance, and metastasis of colorectal cancer (CRC), but the underlying mechanism has seldom been reported. Herein, our study aimed to reveal an exosomal miRNA-mRNA network involved in CRC by performing bioinformatical analysis.

**Methods:**

The mRNA and miRNA data of colon adenocarcinoma and rectal adenocarcinoma were downloaded from The Cancer Genome Atlas (TCGA) database, and exosomal miRNAs data were downloaded from the GEO dataset GSE39833. The differential expression analysis was performed using “limma” and “edgeR”. Target mRNAs of miRNAs were predicted using FunRich 3.1.3, miRNAtap and multiMiR. The candidate mRNAs and exosomal miRNAs were obtained by intersecting two groups of differentially expressed miRNAs and intersection of the differential expressed mRNAs and the target mRNAs, respectively. Key mRNAs and exosomal miRNAs were identified by the least absolute shrinkage and selection operator regression analysis, and used to construct the exosomal miRNA-mRNA network. The network verified was by receiver operating characteristic curve, GEPIA and LinkedOmics. Functional enrichment analysis was also performed for studied miRNAs and mRNAs.

**Results:**

A total of 6568 differentially expressed mRNAs and 531 differentially expressed miRNAs from TCGA data, and 166 differentially expressed exosomal miRNAs in GSE39833 dataset were identified. Next, 16 key mRNAs and five key exosomal miRNAs were identified from the 5284 candidate mRNAs and 61 candidate exosomal miRNAs, respectively. The exosomal miRNA-mRNA network with high connectivity contained 13 hub mRNAs (*CBFB*, *CDH3*, *ETV4*, *FOXQ1*, *FUT1*, *GCNT2*, *GRIN2D*, *KIAA1549*, *KRT80*, *LZTS1*, *SLC39A10*, *SPTBN2*, and *ZSWIM4*) and five hub exosomal miRNAs (*hsa-miR-126*, *hsa-miR-139*, *hsa-miR-141*, *hsa-miR-29c*, and *hsa-miR-423*). The functional annotation revealed that these hub mRNAs were mainly involved in the regulation of B cell receptor signaling pathway and glycosphingolipid biosynthesis related pathways. All hub mRNAs and hub exosomal miRNAs exhibited high diagnosis value for CRC. Furthermore, the association of the hub mRNAs with overall survival, stages, and MSI phenotype of CRC revealed their important roles in CRC progression.

**Conclusion:**

This study constructed an exosomal miRNA-mRNA network which may play crucial roles in the carcinogenesis and progression of CRC, thus providing potential diagnostic biomarkers and therapeutic targets for CRC.

## Background

As one of the common malignant tumors, colorectal cancer (CRC) is the third leading cause of cancer death worldwide [1, 2]. In recent years, the rapid increases in both CRC incidence and mortality are becoming a heavy burden in many countries [1]. Accurate early diagnosis can enable CRC patients to receive timely and accurate treatment, thereby reducing CRC mortality. It is common to perform a colonoscopy in patients with bowel symptoms due to the suspicion of CRC, but the value of symptoms as indicators of CRC is poor [3]. Some researchers previously proposed that several biomarkers, including carcinoembryonic antigen (CEA) and calprotectin, might be used as a predictor of CRC for symptomatic patients. However, the relatively low specificity and sensitivity of such markers preclude their use for the early diagnosis of CRC [3, 4]. Biopsy plays a decisive role in early cancer and polyposis detection in CRC, which is also important in the differential diagnosis of cancer. This method has made it possible to determine the nature, histological type, and malignancy of the tumor, and can help determine the prognosis, and guide the clinical treatment. However, it is still unpopular in clinical applications because of its cumbersomeness and the difficulty in obtaining a satisfactory specimen [5]. Therefore, it is necessary to identify accurate biomarkers for CRC treatment to facilitate the accurate early diagnosis of grading tumor stage and lesion, and to direct the treatment in CRC [6].

Exosomes are tiny goblet shaped vesicles, 30–140 nm in diameter, that are secreted by cells including immune cells, nerve cells, stem cells, and tumor cells [7–9]. Increasing number of studies elucidated the correlation between exosome production and tumorigenesis. Tumor-derived exosomes participate in the exchange of genetic information between tumor cells and basal cells, which results in angiogenesis and promotes tumor growth and invasion. Recently, exosomes that contain various RNA and protein components have become the focus of intensive research as tumor markers for diagnosis and treatment [10, 11]. Identifying useful biomarkers in exosomes of CRC for diagnosis, prediction of prognosis, and treatment response have achieved great progress in recent years. It has been demonstrated that several exosomal miRNAs, such as *miR-125a-3p* [12] and *miR-638* [13], might be useful as diagnostic biomarkers in CRC. Besides, Ogata-Kawata, Izumiya [14] suggested that seven exosomal miRNAs including *miR-223*, *let-7a*, and *miR-150* were significantly related to CRC, and most of these miRNAs showed a higher sensitivity for detection of CRC than CEA. All the evidence described above highlights the important role of exosomal miRNA in CRC, as this has become a promising field for finding CRC biomarkers. Although the miRNA-driven mechanisms of cancer have been investigated through many studies, their molecular mechanisms remain unclear and there are still many unidentified exosomal miRNAs associated with tumors. Therefore, further in-depth studies are needed to identify exosomal miRNAs that may serve as potentially effective targets in CRC diagnosis and treatment. Meanwhile, the exploration of regulatory networks between exosomal miRNAs and mRNAs is beneficial for comprehensively understanding the molecular mechanism of CRC development.

Rapid technological advancements in the big data era have spurred the development of bioinformatics into a fast-growing field with applications in a wide range of areas including medical research. Bioinformatics analysis based on high-throughput platforms is an efficient and promising tool to identify biomarkers for diagnosis and prognosis of various cancers [15, 16]. Exploring the interactions of genetic components is essential to understand the mechanisms of carcinogenesis and tumor progression. Scholars previously established and validated a miRNA-mRNA network to reveal the regulatory mechanisms of miRNA in CRC [17]. Besides, Li et al. [18] uncovered a miRNA-regulatory network, and identified two key miRNAs which exhibited dominant regulatory activities in malignant progression of glioma. However, the number of genes exceeds the number of individuals in most datasets, making the interactions discovered by conventional data analytical approaches unreliable. To deal with this issue, one of machine learning approaches, the least absolute shrinkage and selection operator (LASSO) has been increasingly applied to identify the genes correlated with tumor progression and estimate the coefficients in the Cox model [19, 20]. After determining the value of tuning parameter by a cross-validation, the LASSO shrinks most of the coefficients towards zero according to tuning parameter, thereby maximizing the out-of-data prediction accuracy.

In this study, based on TCGA and GSE39833 datasets, differentially expressed exosomal miRNAs were identified by integrating multiple bioinformatics analysis methods. The LASSO approach was subsequently conducted for the identification of key mRNAs and key exosomal miRNAs. An online prediction database, microRNA Data Integration Portal (miRDIP) v4.1 was used to obtain interactions with high confidence class between exosomal key miRNAs and target mRNAs. After importing the interactions into Cytoscape, an exosomal miRNA-mRNA network consisting of 13 mRNAs and five exosomal miRNAs was obtained. The receiver operating characteristic (ROC) curve analysis was applied to validate the diagnostic efficacy of RNAs from the network for CRC. Further analyses uncovered the roles of these 13 mRNAs in CRC progression. Our findings serve as a valuable resource to further explore the mechanisms of CRC development and progression, and provide potentially effective diagnostic markers and therapeutic targets for CRC.

## Methods

**Fig. 1:**
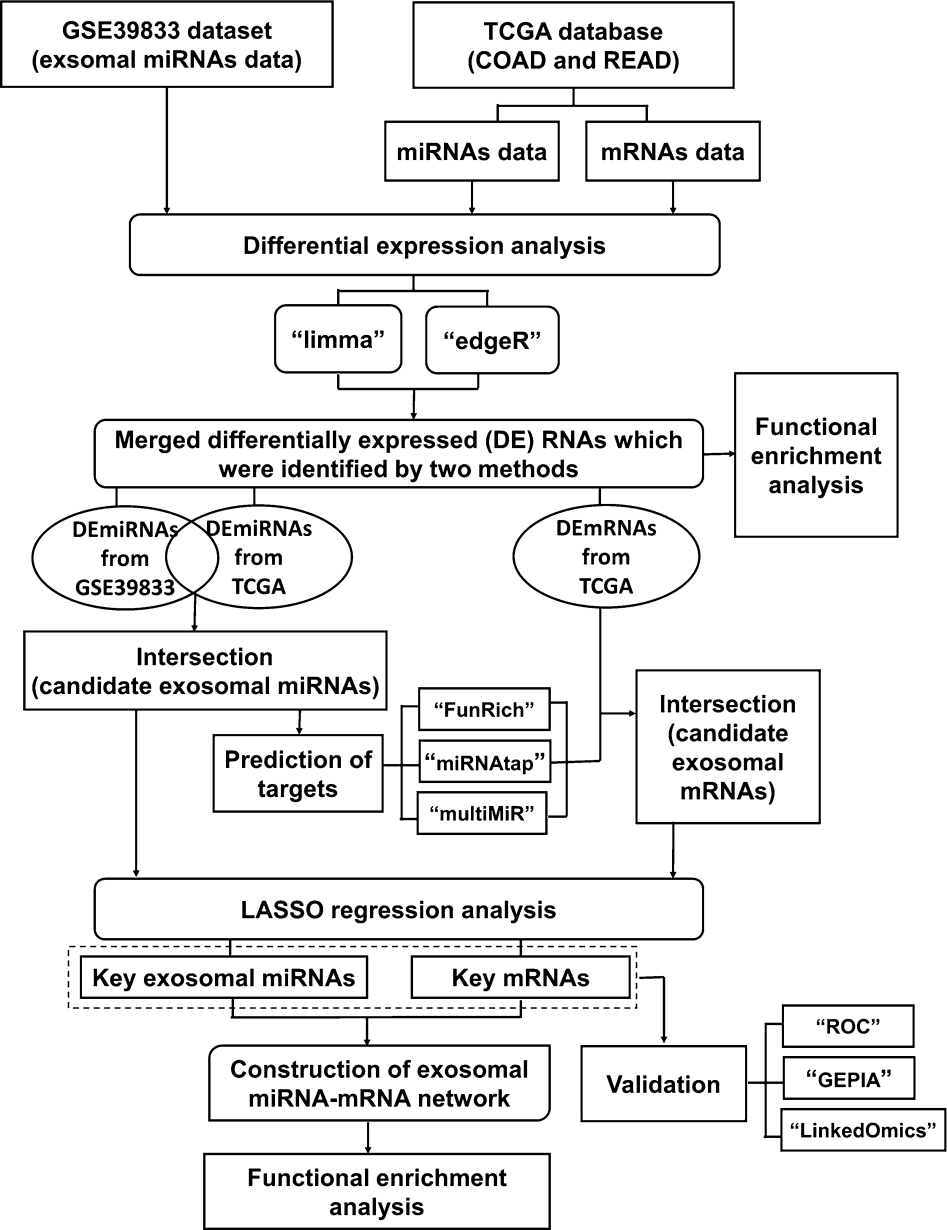
The flowchart of this study

The workflow of this study was described in Fig. 1.

### Data collection

CRC exosomal miRNA dataset GSE39833 was acquired from the GEO database (https://www.ncbi.nlm.nih.gov/geo/); exosomal miRNAs were collected from the sera of 88 colon cancer patients and 11 healthy controls [14]. The dataset contained 15,739 probe IDs. The mRNA and miRNA data of colon adenocarcinoma (COAD) and rectal adenocarcinoma (READ) datasets were downloaded from the cancer genome database (TCGA) (https://portal.gdc.cancer.gov). The TCGA analyze_Normalization function from R package “TCGAbiolinks” was used to filter out extremely lowly expressed genes in TCGA RNA-sequencing data.

### Differential expression analysis (DEA)

Using R-packages limma and edgeR [21], we performed the DEA with threshold parameters defined as |log2FC| > 1 and FDR < 0.05 (FC: fold change, FDR: false discovery rate) to identify the differentially expressed mRNAs and miRNAs related to both COAD and READ in TCGA database. In the meanwhile, the differentially expressed exosomal miRNAs were identified based on the GSE39833 dataset. The differentially expressed mRNAs and miRNAs identified by limma and edgeR were visualized using volcano plots, respectively. The results for the two types of DEA methods from the same data were directly merged before conducting subsequent studies.

### Prediction of target mRNAs for exosomal miRNAs

The differentially expressed miRNAs identified from TCGA data and GSE39833 dataset were intersected to obtain the candidate exosomal miRNAs that were closely related to CRC. The target mRNAs of the candidate exosomal miRNAs were predicted by using FunRich 3.1.3, and R-packages miRNAtap and multiMiR, respectively. After merging the target mRNAs predicted by these three tools, target mRNAs potentially involved in regulation of CRC progression by exosomal miRNAs (our study defined these mRNAs as candidate mRNAs) were acquired by intersection of the differentially expressed mRNAs and target mRNAs.

### Identification of key mRNAs and exosomal miRNAs

As a powerful algorithm, LASSO is used for extracting relevant factors from a large number of unrelated factors. Thus, the LASSO approach was applied to identify the key mRNAs and key exosomal miRNAs that are closely related to CRC from the large number of candidate exosomal miRNAs and candidate mRNAs with the contribution of each RNA being weighted using relative coefficients. The optimal number (λ) of key mRNA and key exosomal miRNAs in combination was determined based on the minimum cross-validated error.

It should be mentioned that, to resolve the data imbalance problem that the number of control samples (n) were obviously less than that of tumor (m), the feature selection was performed multiple times in this study. Each time, n samples were randomly extracted from m tumor samples, and subsequently combined with all of the control samples (n) as the training sets for feature selection. Finally, the times that all features have been screened out in the feature selection were counted, those features with high counts were considered as key features [22]. In this study, LASSO algorithm was performed as the feature selection method, and ten-fold cross validation was used to evaluate the robustness of the feature and parameter λ. The selections of candidate exosomal miRNAs and candidate mRNAs were performed a thousand and ten thousand times, respectively. The candidate exosomal miRNAs and mRNAs were considered as the key exosomal miRNAs and their target mRNAs, respectively, when counts of their occurrences in LASSO regression results were more than 500 and 5000.

### Exosomal miRNA-mRNA network model construction

By using miRDIP v4.1 online tool, the interactions between the key mRNAs and key exosomal miRNAs were obtained. Then, the interactions showing high confidence class were chosen for construction of exosomal miRNA-mRNA network by using Cytoscape v3.7.2. The mRNAs in the network were considered as hub mRNAs in this study.

### Validation of the hub mRNAs and key exosomal miRNAs

The ROC curve analysis was applied to validate the hub mRNAs and key exosomal miRNAs. GEPIA (http://gepia.cancer-pku.cn/) is a web portal for analyzing cancer type and multiple gene data. It provides easy access to public cancer type and gene data and allows users to use advanced computers to validate biomarkers or potential genes of interest. The overall survival of the key mRNAs was performed using GEPIA. The expression levels of the mRNAs at different stages of CRC were then conducted. Besides, the LinkedOmics (http://www.linkedomics.org/admin.php) was applied to perform the expression levels of the hub mRNAs in microsatellite instability (MSI) phenotype of CRC.

## Results

### Differential expression analysis of the mRNA and miRNA in COAD and READ

**Fig. 2:**
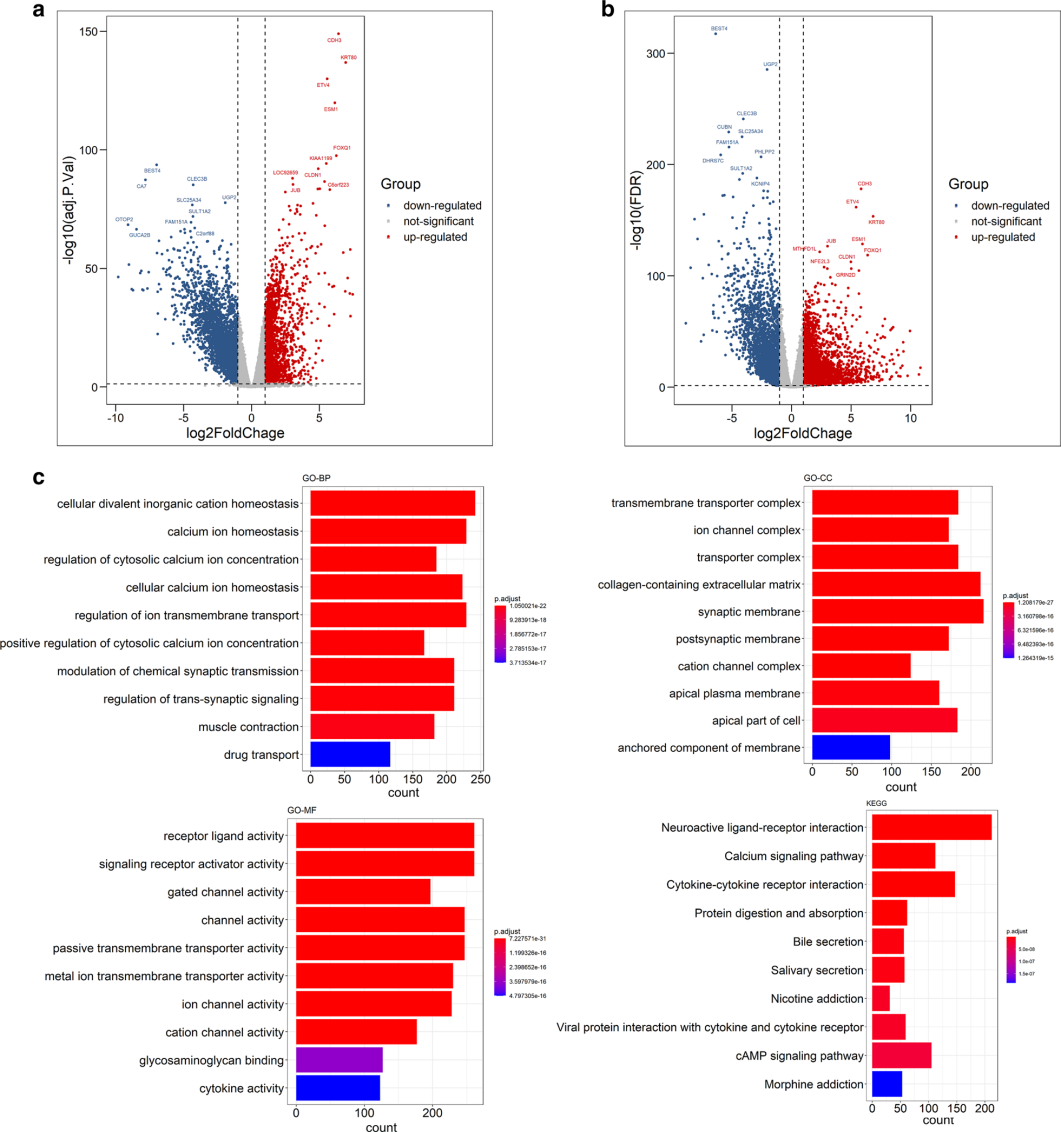
Results of differentially expression analysis in mRNAs from TCGA. The volcano plots of the differentially expressed mRNAs identified by **a** “limma” and **b** “edgeR”, respectively. **c** The functional enrichment analysis of differentially expressed mRNAs obtained by merging the results of “limma” and “edgeR”

**Fig. 3:**
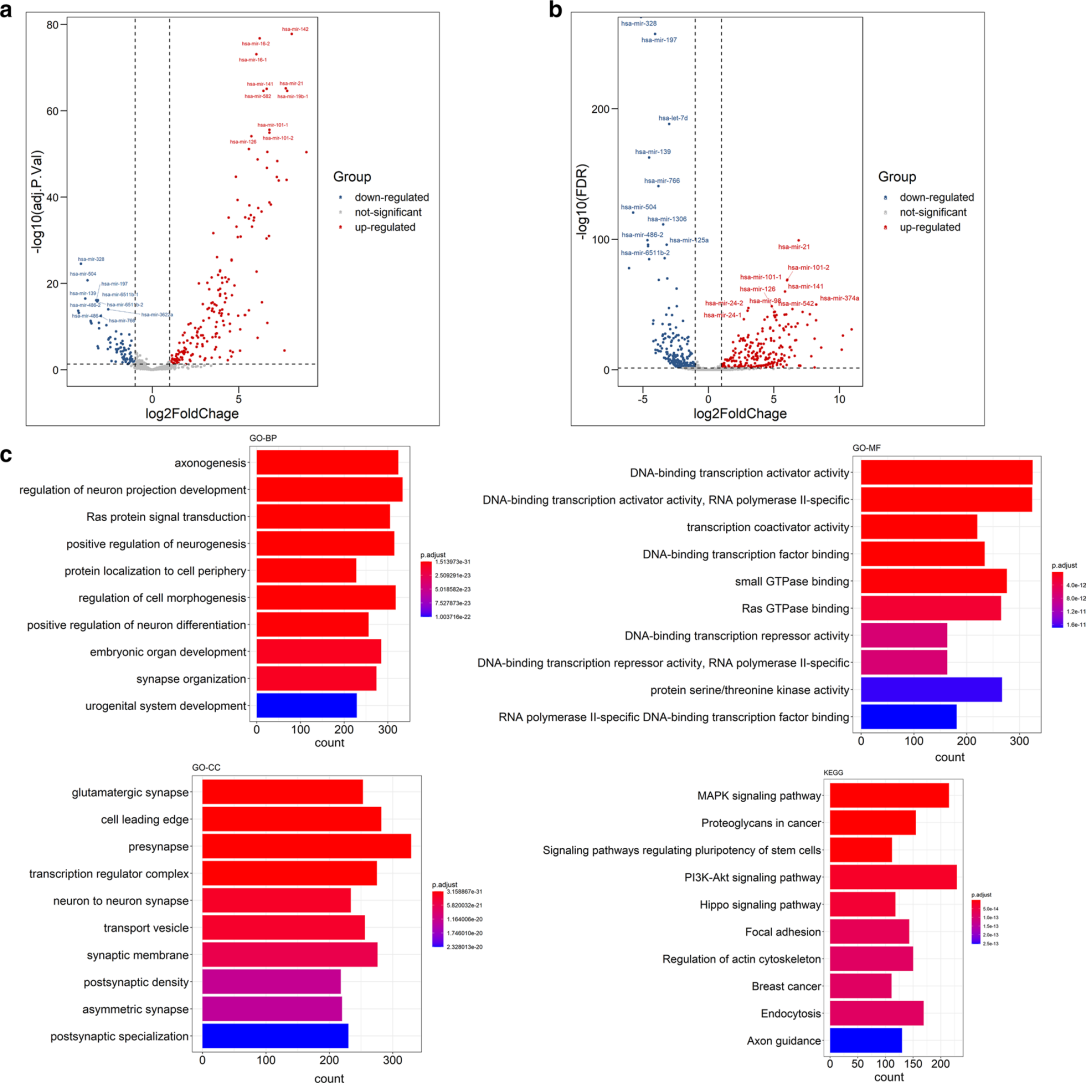
Results of differentially expression analysis of miRNAs from TCGA. The volcano plots of the differentially expressed mRNAs identified by **a** “limma” and **b** “edgeR”, respectively. **c** The functional enrichment analysis of differentially expressed miRNAs obtained by merging the results of “limma” and “edgeR”

**Fig. 4:**
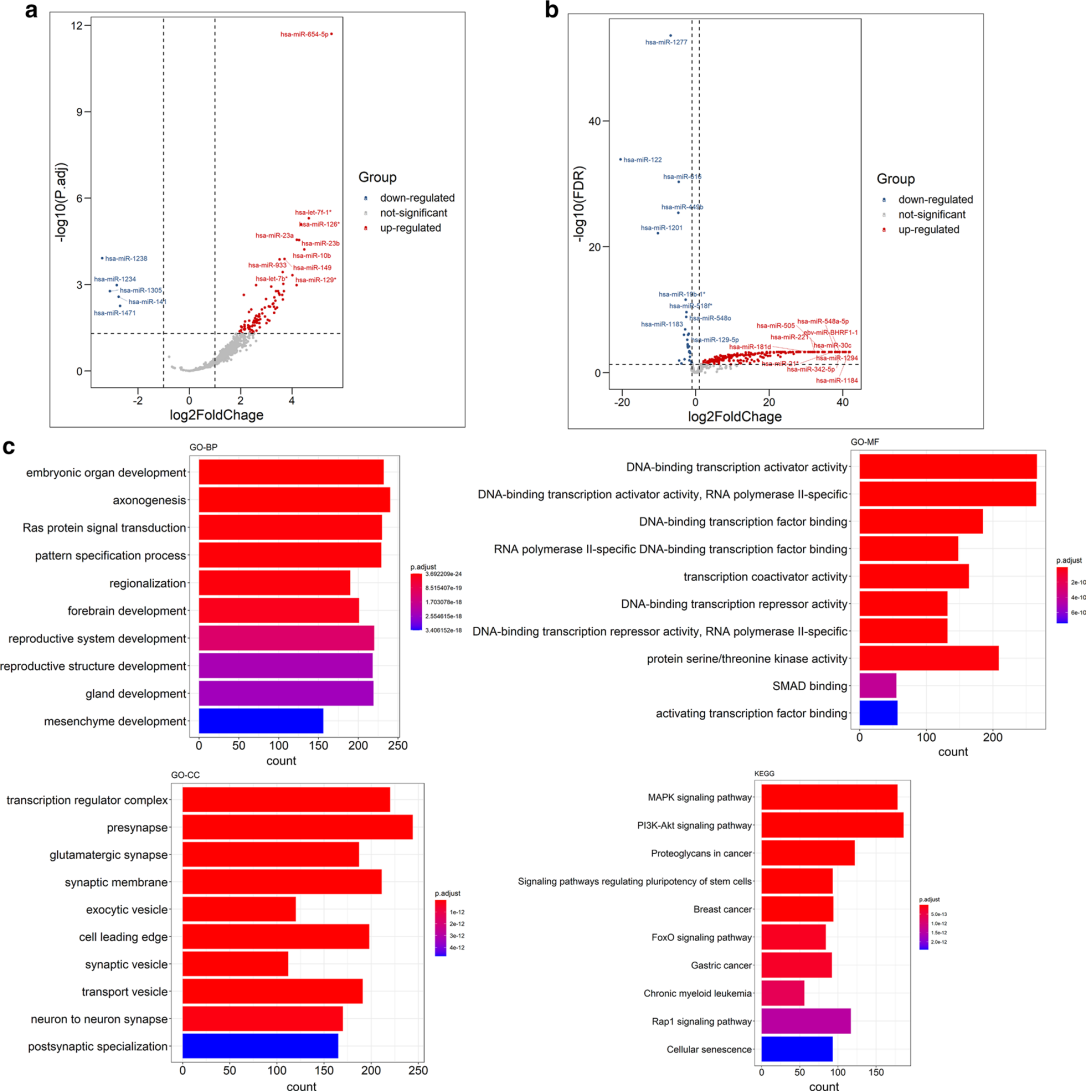
Results of differentially expression analysis in exosomal miRNAs from GSE39833 dataset. The volcano plots of the differentially expressed exosomal miRNAs identified by **a** “limma” and **b** “edgeR”, respectively. **c** The functional enrichment analysis of differentially expressed exosomal miRNAs obtained by merging the results of “limma” and “edgeR”

To identify the differentially expressed mRNAs and miRNAs in COAD and READ, and differentially expressed exosomal miRNA between CRC patients and healthy individuals, we performed DEA using the R packages of “limma” and “edgeR”. The results were shown in Table 1. The differentially expressed mRNA and miRNA were visualized by volcano plots. The volcano plots for the result of “limma” were displayed in Figs. 2a, 3a and 4a, while those for the result of “edgeR” were displayed in Figs. 2b, 3b and 4b. After merging the results of “limma” and “edgeR”, a total of 6567 differentially expressed mRNAs, 530 differentially expressed miRNAs, and 165 differentially expressed exosomal miRNAs were obtained. The identified differentially expressed mRNAs and miRNAs were listed in Additional file 1: Table S1.

**Table 1:** Results of differentially expressed mRNAs and miRNAs from TCGA, and exosomal miRNAs of GSE39833

Compare	Style	Identification method	Log_2_FC_Cutoff	padj_Cutoff	All differential expressed Num	Up regulated Num	Down regulated Num	Total Num after merging
Tumor versus normal (TCGA-COADREAD)	mRNA	limma	1	0.05	5621	2585	3036	6567
		edgeR	1	0.05	5921	2881	3040	
Tumor versus normal (TCGA-COADREAD)	miRNA	limma	1	0.05	476	161	315	530
		edgeR	1	0.05	502	251	251	
Tumor versus normal (GSE39833)	Exosomal miRNA	limma	1	0.05	79	38	41	165
		edgeR	1	0.05	274	244	30

The functional enrichment analysis of the differentially expressed mRNAs revealed that they were mainly involved in processes such as cellular divalent inorganic cation homeostasis and calcium ion homeostasis in biological process (GO-BP), transmembrane transporter/ion channel complex and collagen-containing extracellular matrix in cellular component (GO-CC), and channel activity, passive transmembrane transporter activity and receptor ligand activity in molecular function (GO-MF). The Kyoto Encyclopedia of Genes and Genomes (KEGG) pathway analysis revealed the interaction of neuroactive ligand-receptor, cytokine-cytokine receptor, and the signaling pathway of calcium and cAMP (Fig. 2c).

The target genes of differentially expressed miRNAs were mainly enriched in processes such as signal transduction of Ras protein, axonogenesis and embryonic organ development in GO-BP; the presynapse, cell leading edge, actin cytoskeleton, and transcription regulator complex in GO-CC; DNA-binding transcription related activities and small/Ras GTPase binding in GO-MF. The KEGG pathway analysis revealed the involvement of genes related to the proteoglycans in cancer, PI3k-Akt and MAPK signaling pathway (Fig. 3c).

As shown in Fig. 4c, the target genes of the differentially expressed exosomal miRNAs were mainly enriched in embryonic organ development and Ras protein signal transduction in GO-BP, presynapse and transcription factor complex in GO-CC, DNA-binding transcription activator activity and DNA-binding transcription activator activity, RNA polymerase II-specific in GO-MF, and proteoglycans in cancer, PI3k-Akt and MAPK signaling pathways in KEGG pathway analysis.

### Screening of candidate mRNAs and exosomal miRNAs

To identify exosomal RNAs involved in CRC development, we performed overlap analysis. After merging the differentially expressed exosomal miRNA data (GSE39833) and the differentially expressed miRNAs in TCGA, we found 61 common miRNAs that showed a consensus between the two sets of miRNA data (Table 2). These 61 miRNAs were considered as candidate exosomal miRNAs. After predicting target mRNAs for the 61 candidate exosomal miRNAs, a total of 18,641 target mRNAs were obtained (Additional file 2: Table S2). Finally, a total of 5284 candidate mRNAs were acquired by intersection of these target mRNAs with the differentially expressed mRNAs.

**Table 2:** The results of the consistent miRNAs between differentially expressed miRNA and exosomal miRNAs

MicroRNA symbol	MicroRNA symbol	MicroRNA symbol
hsa-let-7c	hsa-mir-150	hsa-mir-337
hsa-let-7d	hsa-mir-1539	hsa-mir-362
hsa-mir-100	hsa-mir-181d	hsa-mir-421
hsa-mir-106b	hsa-mir-192	hsa-mir-423
hsa-mir-10a	hsa-mir-193b	hsa-mir-424
hsa-mir-10b	hsa-mir-197	hsa-mir-484
hsa-mir-1180	hsa-mir-206	hsa-mir-495
hsa-mir-1224	hsa-mir-22	hsa-mir-539
hsa-mir-1227	hsa-mir-221	hsa-mir-545
hsa-mir-1228	hsa-mir-224	hsa-mir-552
hsa-mir-1229	hsa-mir-23a	hsa-mir-574
hsa-mir-1234	hsa-mir-23b	hsa-mir-605
hsa-mir-1237	hsa-mir-27a	hsa-mir-654
hsa-mir-1238	hsa-mir-27b	hsa-mir-671
hsa-mir-126	hsa-mir-296	hsa-mir-760
hsa-mir-1306	hsa-mir-29a	hsa-mir-766
hsa-mir-1307	hsa-mir-29c	hsa-mir-770
hsa-mir-130a	hsa-mir-301a	hsa-mir-92b
hsa-mir-139	hsa-mir-30a	hsa-mir-95
hsa-mir-141	hsa-mir-30b	
hsa-mir-149	hsa-mir-328	

There were 61 efficient miRNAs in exosomes

### Construction of exosomal miRNA-mRNA network

**Fig. 5:**
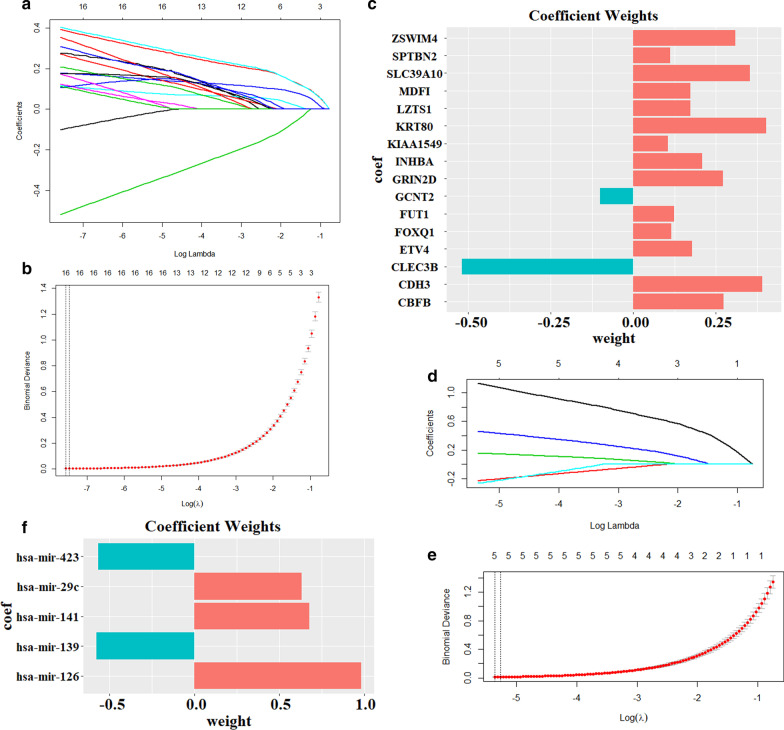
Identification of key mRNAs and exosomal miRNAs by LASSO regression approach. **a** Tuning parameter of LASSO model selection, **b** LASSO coefficient profiles, and **c** coefficient values for the 16 key mRNAs. **d** Tuning parameter of LASSO model selection, **e** LASSO coefficient profiles, and **f** coefficient values for the five key exosomal miRNAs

The path coefficient and cross-validation binomial deviance curve for logistic LASSO were carried out to minimize overfitting in the models of mRNAs or miRNAs, and the optimal lambda of mRNA model (λ = 16) and miRNA model (λ = 5) were subsequently obtained (Fig. 5a, b, d, e). A total of 16 key mRNAs and five key exosomal miRNAs were identified, and their coefficient weights were depicted in Fig. 5c, f, respectively. By using the bidirectional search function in the mirDIP database, 25 bidirectional relationships with high confidence class were obtained after entering the key mRNAs and exosomal miRNAs.

**Fig. 6:**
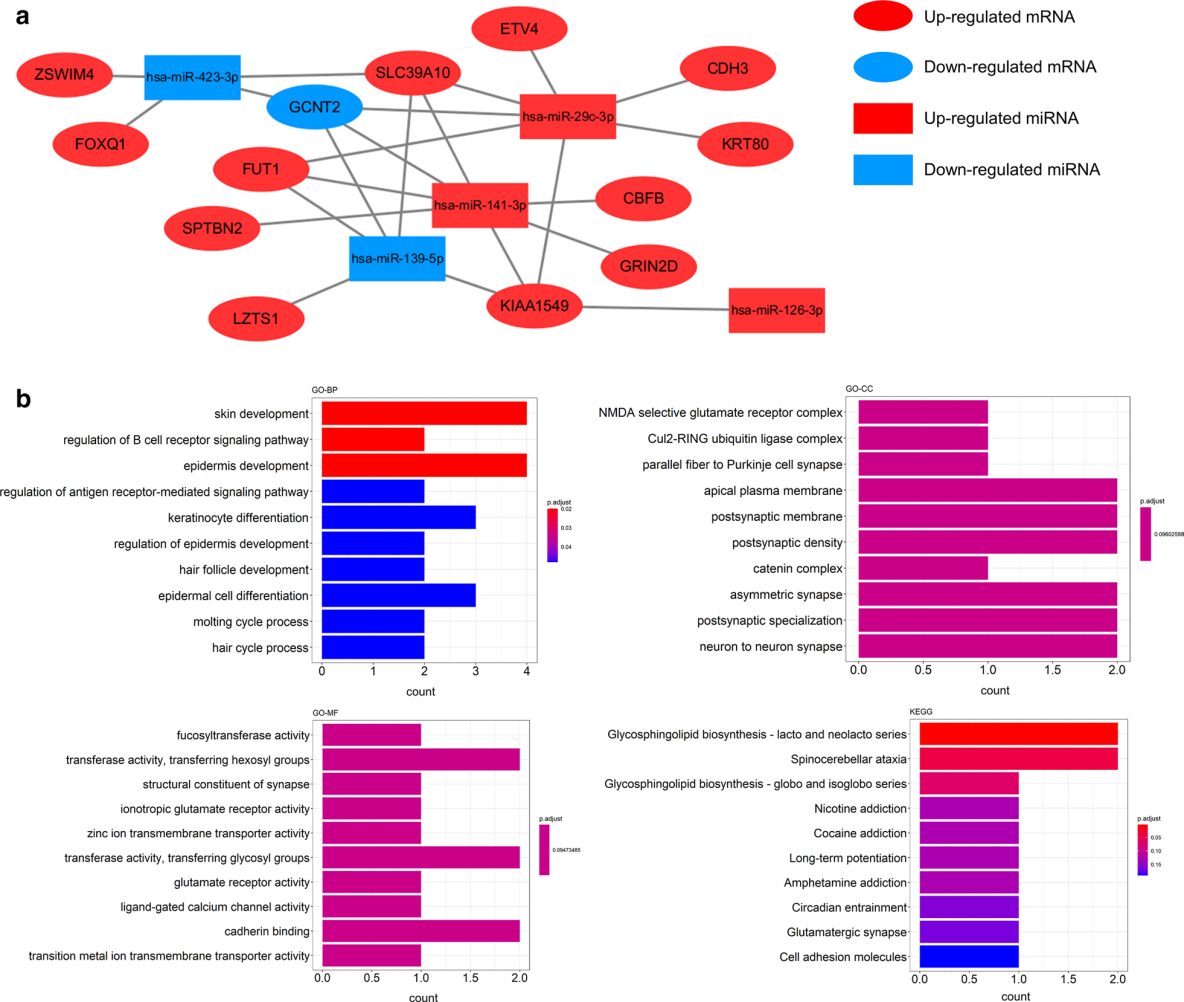
Analysis of hub mRNAs/exosomal miRNAs. **a** Exosomal miRNA-mRNA network. **b** Functional enrichment analysis of the hub mRNAs

As shown in Fig. 6a, an exosomal miRNA-mRNA network consisted of five miRNAs (*hsa-miR-126*, *hsa-miR-139*, *hsa-miR-141*, *hsa-miR-29*c, and *hsa-miR-423*) and 13 mRNAs (*CBFB*, *CDH3*, *ETV4*, *FOXQ1*, *FUT1*, *GCNT2*, *GRIN2D*, *KIAA1549*, *KRT80*, *LZTS1*, *SLC39A10*, *SPTBN2*, and *ZSWIM4*) was constructed. These mRNAs and miRNAs were respectively considered to be hub mRNAs and hub exosomal miRNAs that might play crucial roles in CRC development via exosomes. According to the network, we found that the *hsa-miR-141* and *hsa-miR-29c* interacted with more than half of mRNAs. Meanwhile, *FUT1*, *GCNT2*, *KIAA1549* and *SLC39A10* were connected with four of five exosomal hub miRNAs. These findings suggested *hsa-miR-141*, *hsa-miR-29c*, *FUT1*, *GCNT2*, *KIAA1549* and *SLC39A10* might be key contributors in the development of CRC. Additionally, the interactions of these miRNA/mRNA also might provide novel insights into the molecular mechanism of the roles of exosomes in CRC. As shown in Fig. 6b, the functional annotation of the 13 hub mRNAs showed that they were mainly enriched in skin development and regulation of B cell receptor signaling pathway in GO-BP, apical plasma membrane and postsynaptic membrane in GO-CC, transferase activity, transferring hexosyl/glycosyl groups in GO-MF, and glycosphingolipid biosynthesis related signaling pathways and Nicotine/Cocaine addiction in the KEGG pathway analysis.

### Validation of the hub mRNAs and hub exosomal miRNAs

**Fig. 7:**
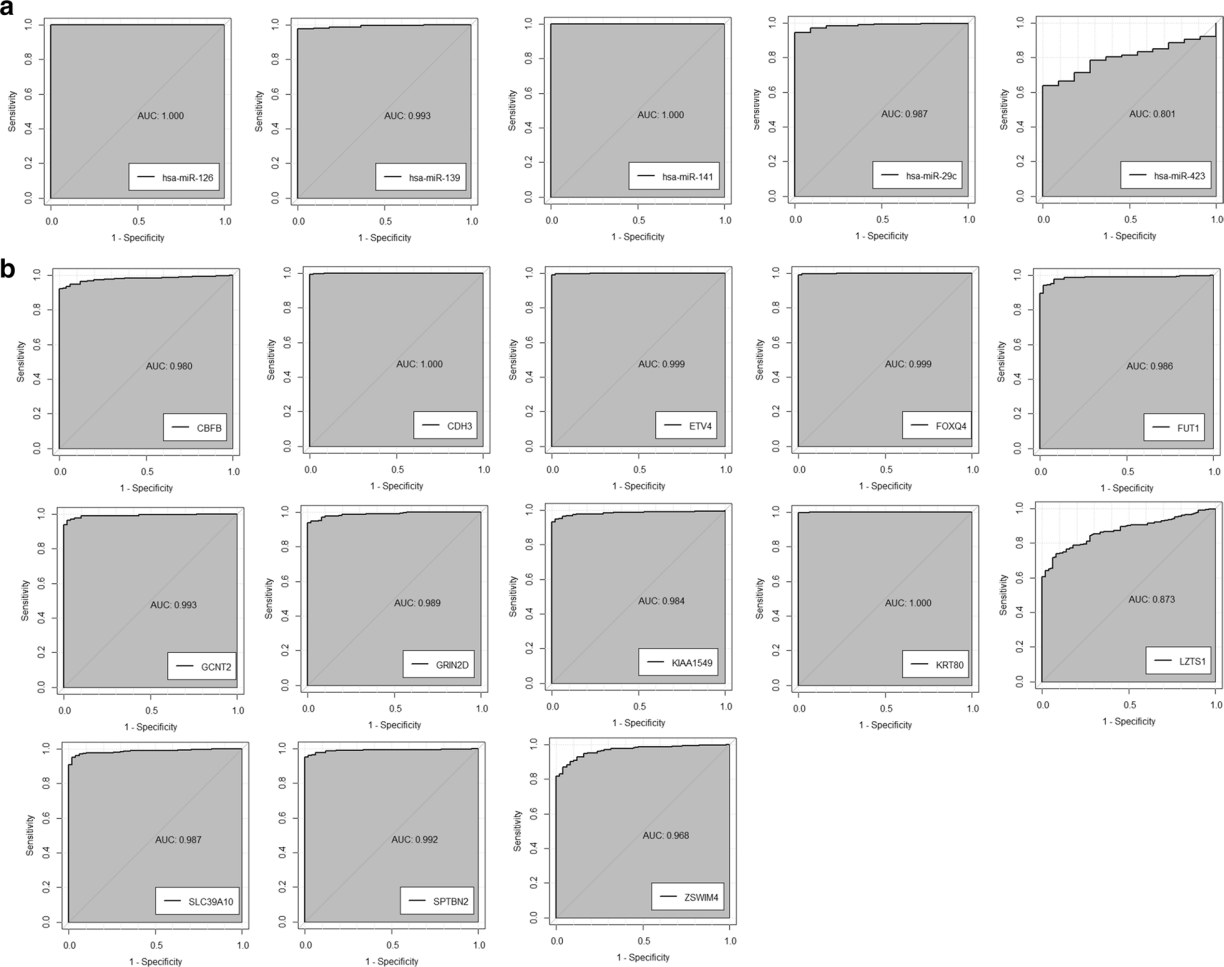
ROC curve analysis of the **a** hub mRNAs and **b** exosomal hub miRNAs

We explored the value of the hub mRNAs and hub exosomal miRNAs as diagnosis biomarkers in CRC by performing ROC curves and calculating the area under the curves (AUCs) [95% confidence intervals (CIs)]. As shown in Fig. 7a, the AUCs of *hsa-miR-126*, *hsa-miR-139*, *hsa-miR-141*, *hsa-miR-29c*, and *hsa-miR-423* were respectively 1.000, 0.993, 1.000, 0.987 and 0.801, which proved that the five hub exosomal miRNAs can well distinguish tumor and normal samples. As shown in Fig. 7b, the AUCs of *CBFB*, *CDH3*, *ETV4*, *FOXQ1*, *FUT1*, *GCNT2*, *GRIN2D*, *KIAA1549*, *KRT80*, *LZTS1*, *SLC39A10*, *SPTBN2*, and *ZSWIM4* were respectively 0.980, 1.000, 0.999, 0.999, 0.986, 0.993, 0.989, 0.984, 1.000, 0.873, 0.987, 0.992, 0.968, and 0.904, suggesting that these hub mRNAs had highly diagnostic accuracy potential to distinguish tumors from normal tissues.

**Fig. 8:**
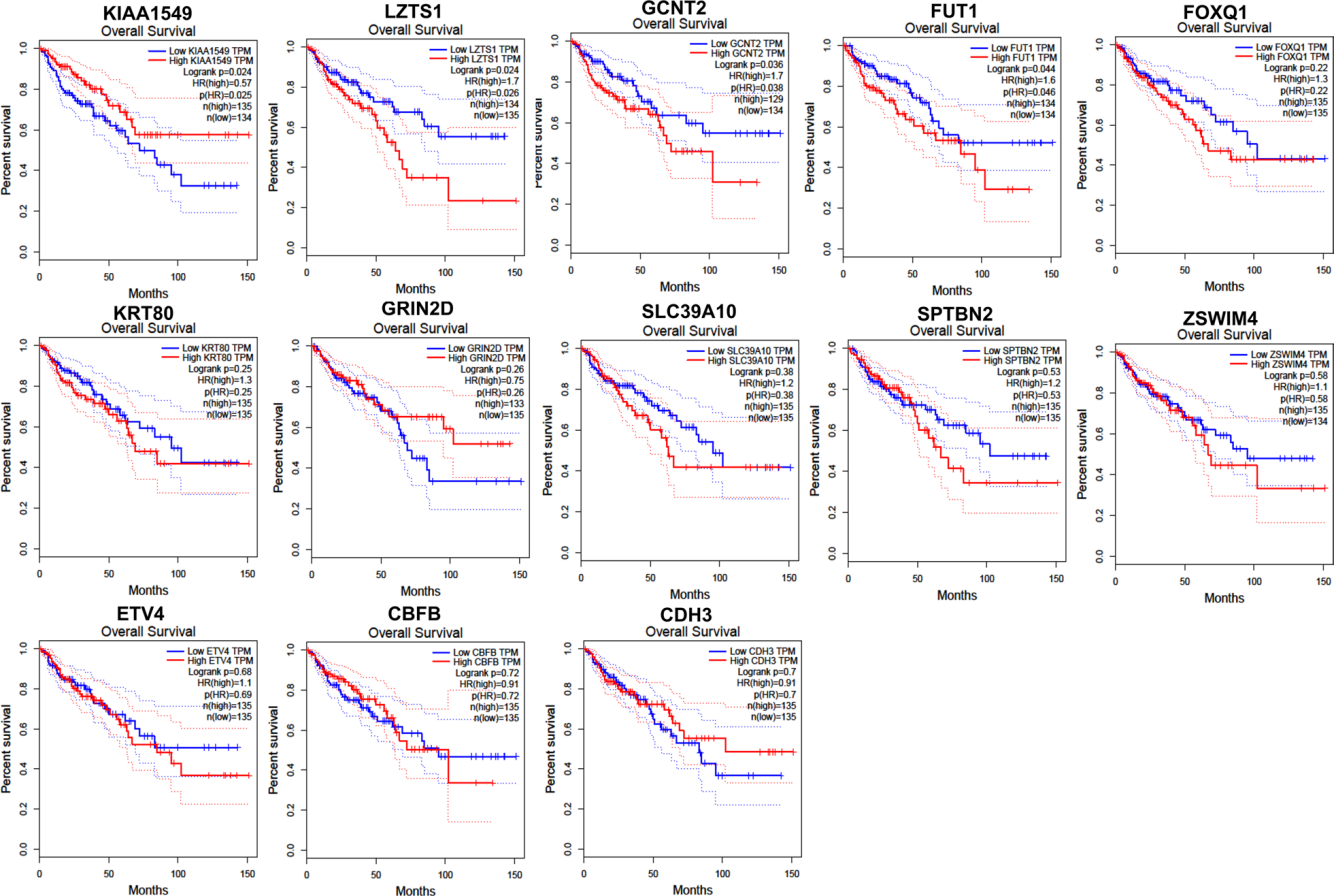
Results for the overall survival (OS) analysis of the hub mRNAs in COAD (Kaplan–Meier Plotter). *Note* The dotted lines on the Kaplan–Meier curves represented 95% confidence intervals

**Fig. 9:**
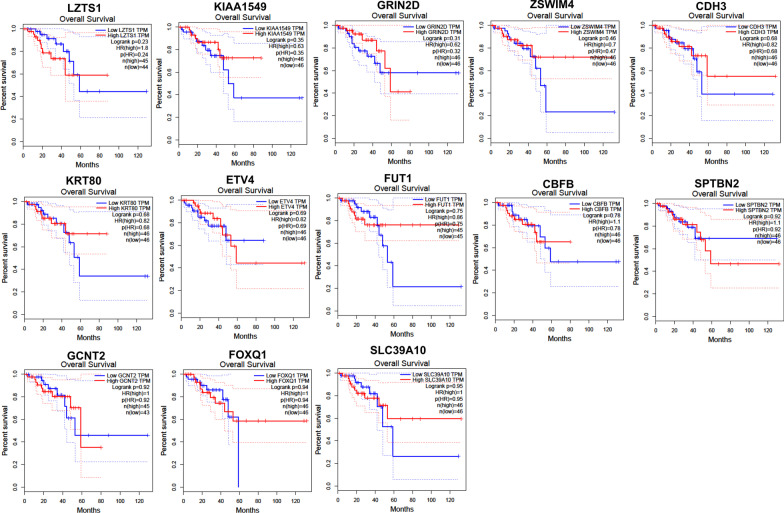
Results for the overall survival (OS) analysis of the hub mRNAs in READ (Kaplan–Meier Plotter). *Note* The dotted lines on the Kaplan–Meier curves represented 95% confidence intervals

**Fig. 10:**
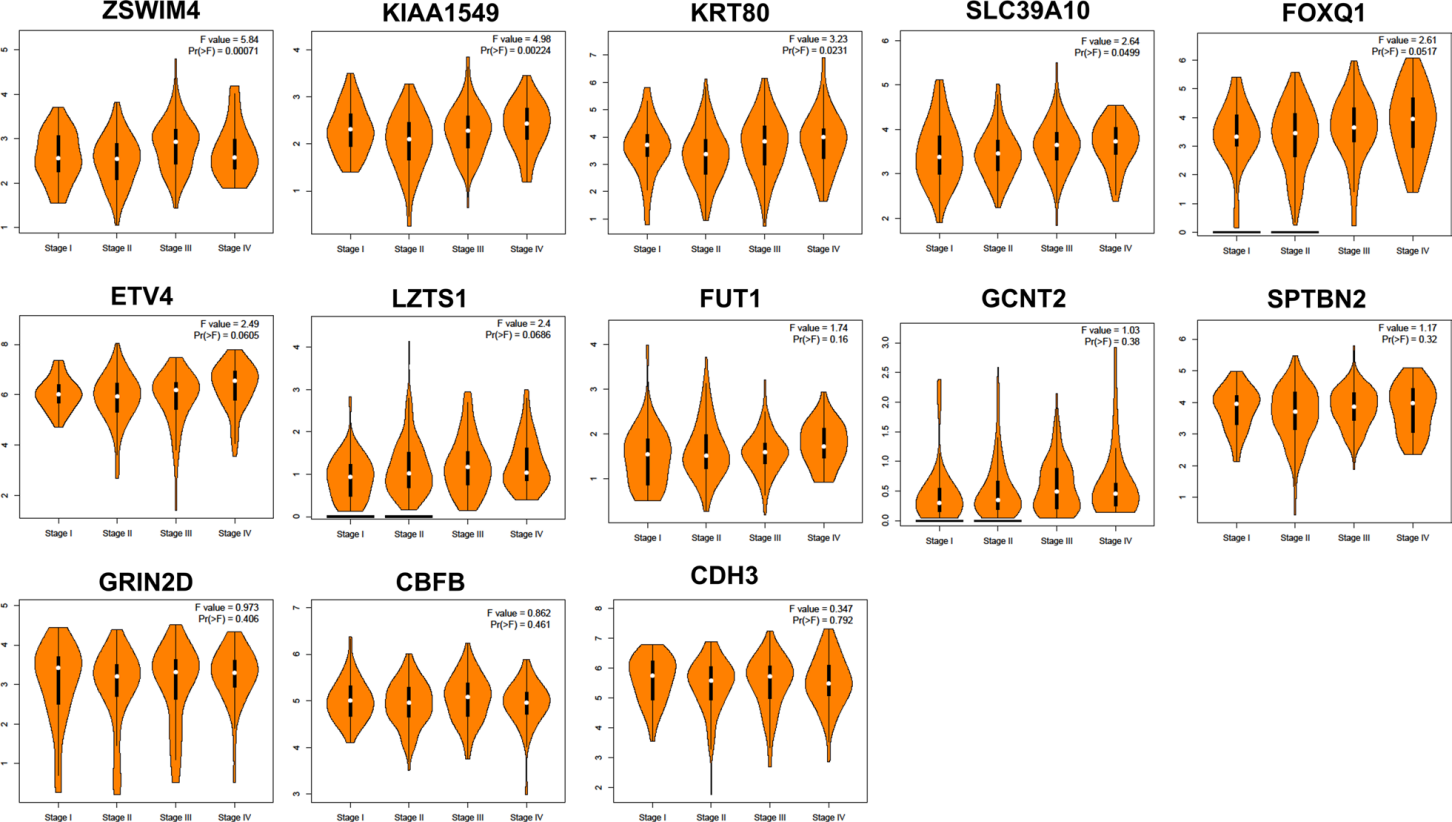
The expression of the hub mRNAs in different tumor stage of COAD patients

**Fig. 11:**
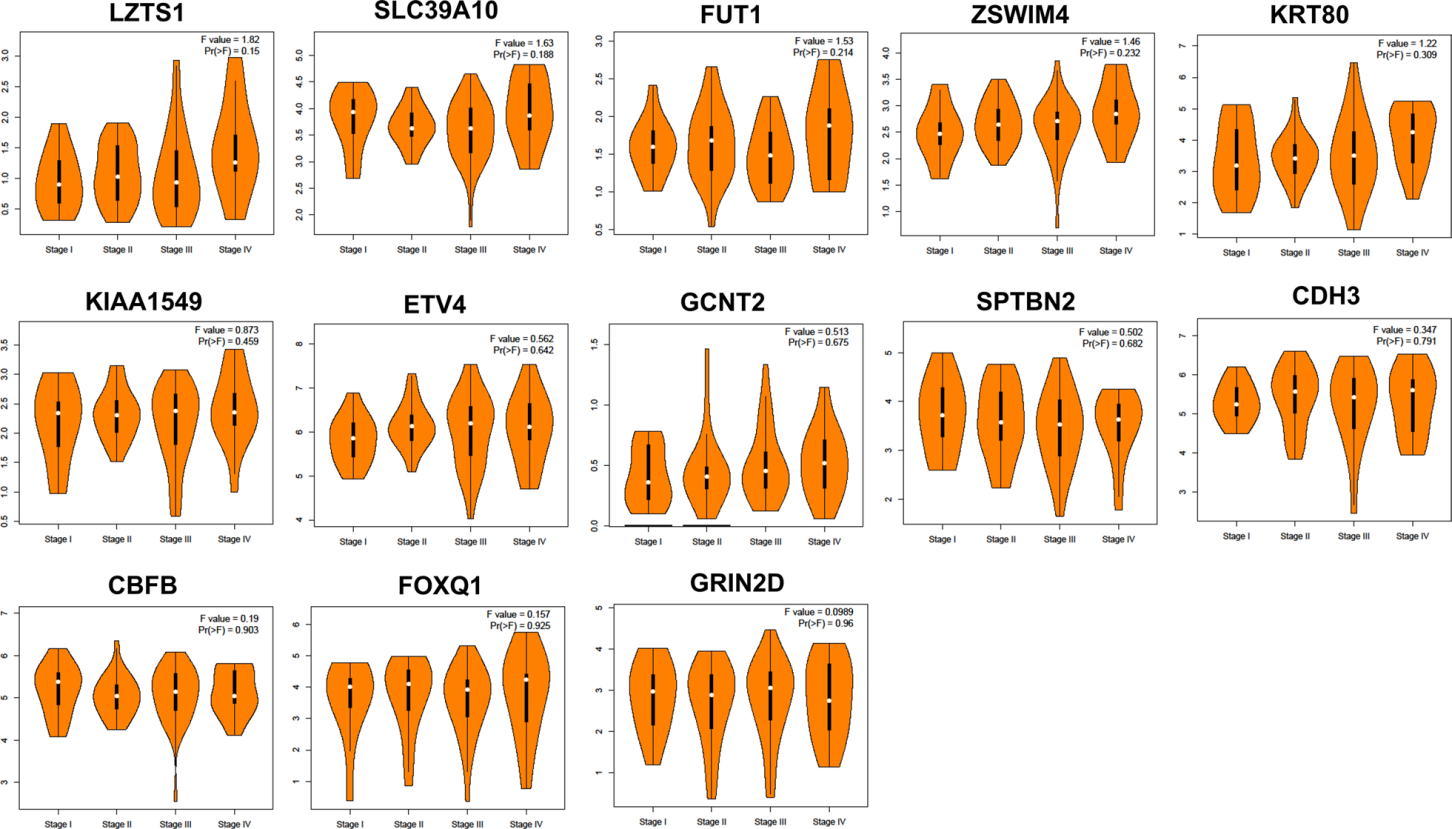
The expression of the hub mRNAs in different tumor stage of READ patients

Additionally, we used GEPIA to conduct the survival and stage analyses of the hub mRNAs. The results showed that the expressions of *FUT1*, *GCNT2*, *KIAA1549* and *LZTS1* were significantly related to the overall survival (OS) in COAD (*P* < 0.05, Fig. 8). All the hub mRNAs were not related to the OS in READ (*P* > 0.05, Fig. 9). Besides, we found a strong correlation of the COAD stages with expressions of *KIAA1549*, *KRT80*, *SLC39A10* and *ZSWIM4* (*P* < 0.05, Fig. 10), hinting that these four hub mRNAs might be involved in the COAD stages. Notably, the stage analysis of the hub mRNAs in READ showed a similar result with the survival analysis in READ (Fig. 11). We speculated that it is because the sample size of READ was small in the TCGA database.

**Fig. 12:**
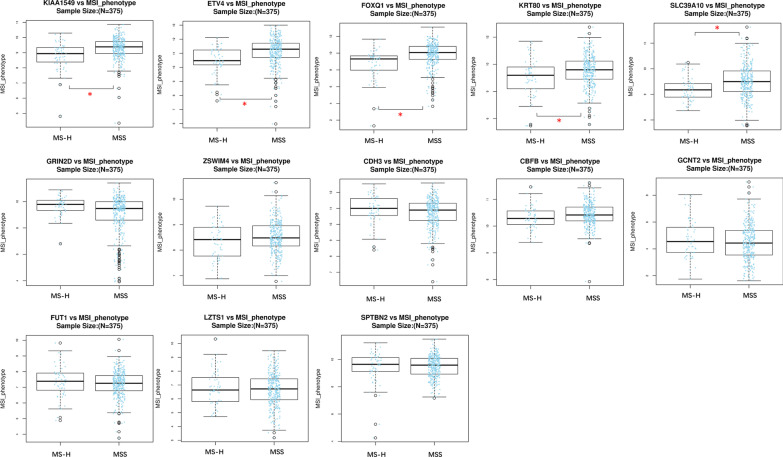
The expression of the hub mRNAs in different MSI phenotype of CRC patients. The expression of *ETV4*, *FOXQ1*, *KIAA1549*, *KRT80*, and *SLC39A10* were significant in distinguishing MS-H and MSS phenotype (*P* value < 0.05)

Furthermore, we investigated the relation of these exosomal miRNA-regulated hub mRNAs with the MSI phenotype in CRC. As shown in Fig. 12, the expression levels of *ETV4*, *FOXQ1*, *KIAA1549*, *KRT80*, and *SLC39A10* were significantly different in microsatellite stable (MSS) and microsatellite instability-high (MS-H) subtype CRC.

## Discussion

CRC, including colon and rectal cancer, has become one of the leading causes of cancer in most countries and regions worldwide in the recent decades [23, 24]. Given the high mortality rate of CRC, there is an urgent need for effective prevention and treatment modalities [25]. With the recent advances in the fields of medicine and biotechnology, precise treatment of CRC through gene targeted therapy is a new and efficient potential therapeutic approach for CRC, which requires the identification of marker genes associated with cancer [26]. Multiple studies have proven that exosomes play an important role in cancer development including angiogenesis, drug resistance, and cancer metastasis [27–29]. Therefore, the properties of exosomes are being exploited for diagnostic, prognostic, and therapeutic applications. It was reported that exosome biomarkers can be used for early diagnosis and prognosis of breast cancer [30]. A previous study conducted by Hu and his colleagues demonstrated that exosomal *miR-92a-3p* was significantly associated with metastasis and chemotherapy resistance in CRC, which may be used for the prediction of prognosis in CRC [31]. However, the underlying mechanism of exosome-regulated CRC progression has not been elucidated. In this study, we aimed to reveal an exosomal miRNA-mRNA network that drives the mechanisms of CRC, and identify potential biomarkers for improving the diagnosis accuracy of CRC.

“limma” and “edgeR” are two approaches of DEA relying on different assumptions and techniques, so the results of the two methods are commonly different. There is no standard to judge which methods obtains more credible results. In order to avoid the possibility of excluding some important RNAs due to only one method, this study used both methods to perform DEA and combine the results for subsequent experiments. Through comprehensive analysis of the open access miRNA and mRNA data for CRC from TCGA, we identified 6567 mRNAs and 530 miRNAs that were differentially expressed in COAD and READ. We also identified 165 differentially expressed exosomal miRNAs based on GSE39833 dataset. It is worth mentioning that the differentially expressed miRNAs and exosomal miRNAs we identified were both mainly involved in MAPK and PI3k-Akt signaling pathways. The MAPK signaling pathway functions as a molecular switch for multiple cellular responses during both homeostasis and pathological conditions [32]. Both the MAPK and PI3k-Akt signaling pathways play a critical regulatory role in the development and progression of CRC [33]. These data indicate that the screened exosomal miRNAs were robust and can potentially affect the tumor fate.

With the aim of screening more robust and potential miRNAs that modulated CRC development via exosomes, we performed intersection of differentially expressed miRNAs with differentially expressed exosomal miRNAs for further studies. After finishing the intersection, a total of 61 candidate exosomal miRNAs were obtained to predict their targeted mRNA. Next, 5284 mRNAs were obtained by overlapping the target mRNAs and differentially expressed mRNAs, which could ensure to get the most potential mRNAs associated with both CRC progression and exosomes. Recently, machine learning approaches have gained popularity in medicine [34, 35]. The prowess and flexibility of machine learning methods enable researchers to extract valuable information from increasing biomedical databases. By integrating a logistic regression model and the LASSO method, scholars established a scoring system that could efficiently predict the risk of thyroid malignancy [36]. A previous study also proved that machine-learning methods might be a prior choice in the analysis of high dimensional data when no prior knowledge is available [37]. Based on the candidate mRNAs and exosomal miRNAs, LASSO method identified five key exosomal miRNAs and 16 key mRNAs, which were significantly weighted. By adopting a LASSO regression analysis, we could efficiently identify the regulators closely associated with CRC progression. Then, we found that all the key exosomal miRNAs we identified were reported in previous studies on cancer. Several studies revealed that the *hsa-miR-126* could be used as a biomarker in different cancers, such as non-small-cell lung cancer [38] and pancreatic adenocarcinoma [39]. Besides, *hsa-miR-126* has been proven to be a tumor suppressor that can inhibit the growth of NSCLC cells and enhance the cytotoxicity of targeted agents [40]. Multiple studies indicated the crucial roles of *hsa-miR-139* in CRC progression [41, 42]. Several researches demonstrated that *hsa-miR-139* exhibits tumor-suppressive function by regulating NOTCH-1 in CRC [43, 44]. Notably, in the last year, Zhao et al. [45] successfully constructed a nanoparticle loaded with *hsa-miR-139* which showed tumor targeting activity and antitumor ability in CRC model. Some previous studies revealed that *hsa-miR-141* is closely related to cancer metastasis. For example, in 2015, Li et al. [46] found that the elevated levels of serum exosomal *hsa-miR-141* were significantly correlated with metastasis of prostate cancer. Besides, Yan et al. [47] demonstrated that *hsa-miR-141* plays a certain role in colon cancer metastasis. Additionally, Li et al. [48] indicated overexpression of *hsa-miR-423* could promote CRC cell proliferation, pointing out a critical role of *hsa-miR-423* on CRC development. All these five exosomal miRNAs exhibited high AUCs in ROC curve analysis, suggesting that they could be used as diagnostic markers for CRC.

As known, exosomes contain a variety of small molecules of RNAs, such as miRNAs and long non-coding RNAs, which could be delivered into the target cells to play certain functions. It is documented that these exosomes of RNAs could participate in complex cell-to-cell communications by changing the gene expression in recipient cells [49, 50]. In multiple diseases, serum exosomes transfer miRNAs into the cell of diseased tissue to modulate the disease development. Thus, we used the mRNA from CRC tissue and miRNAs from serum exosomes to establish an exosomal miRNA-mRNA network for understanding the molecular mechanism of exosomal miRNAs in intercellular communications under CRC microenvironment. Afterward, a total of 13 hub mRNAs regulated by exosomal miRNAs were identified for further analyses and validation. Functional enrichment analysis of these mRNAs revealed they were mainly involved in the terms of skin development and glycosphingolipid biosynthesis related signaling pathways. As the typical components of animal cell membranes, glycosphingolipids play key roles in cells proliferation, adhesion, motility and differentiation and its abnormal biosynthesis is usually found in various cancers including CRC [51, 52]. Therefore, we speculated that the hub exosomal miRNAs might change the expression of hub mRNAs to regulate glycosphingolipid biosynthesis, thereby affecting proliferation, migration and invasion of CRC cells. Then, we found that *FOXQ1*, *GCNT2* and *KIAA1549* showed the highest interactions (four) with exosomal miRNAs in the network and showed a strong correlation with the survival times of COAD patients. Nakamura et al. [53] revealed that *GCNT2* is closely related to CRC metastasis. *FOXQ1* has been identified as a tumor promoter of CRC in a study conducted by Liu et al. [54]. Cumulative studies indicated that *FOXQ1* is capable of promoting metastasis in diverse cancers [55–57], especially in CRC [54, 58]. Nevertheless, in the current study, the expression of *KIAA1549* was not only significantly associated with OS of COAD patients, but also strongly related to stages and MSI phenotypes in CRC. There is no report that explores the role of *KIAA1549* in cancers. Therefore, we speculated that *KIAA1549* might play a critical role in the mechanism of CRC, and could be a novel and robust target in CRC therapy. *SLC39A10*, another hub mRNA connected with the four exosomal hub miRNAs, encodes zinc transporter ZIP10 which is involved in cell migration during tumor progression [59]. In this study, we found its expression was significantly different in stages of COAD as well as MSI phenotypes of CRC. Apart from *KIAA1549* and *GCNT2*, we found the expression of *FUT1* was also significantly related to survival times of COAD patients. *FUT1* was considered as a potential tumor suppressor, its abnormal expression was frequently found in various cancers [60, 61]. Zhou et al. [62] demonstrated that *LZTS1* could suppress cell proliferation and prohibit tumor growth in CRC by regulating AKT-mTOR signaling pathway. Besides, both the expression level of *KRT80* and *FOXQ1* could efficiently distinguish the phenotype of MSS from MS-H in CRC. The MSI status could alter the tumor microenvironment of CRC patients in different ways, thereby influencing the efficacy of immune checkpoint inhibitors in CRC patients. Several studies revealed that CRC patients with MS-H phenotype were more sensitive to immune checkpoint inhibitors than those with MSS phenotype [63, 64]; nevertheless, the underlying mechanism is still obscure. Our study implied that *SLC39A10*, *KRT80*, *FOXQ1* and *KIAA1549* might be involved in the process of MSI and used to be potential biomarkers of MSI phenotype testing. However, we found that all the identified mRNAs that were regulated by exosomal miRNAs showed no significant correlation with prognosis and stages in READ patients, which is possibly due to the small sample size of READ patients. Furthermore, the ROC analysis revealed that the identified key exosomal miRNAs and hub mRNAs are likely to be good biomarkers with high sensitivity and specificity for diagnosis of CRC in patients. Thus, the detection of the *hsa-miR-126*, *hsa-miR-139*, *hsa-miR-141*, *hsa-miR-29c*, and *hsa-miR-423* in exosomes from patient blood may serve as a non-invasive diagnosis biomarkers for CRC.

Based on our results and above-mentioned literature, we suppose that there are multiple regulatory axes related to CRC development in intracellular communications mediated by serum exosomes. The current study suggested that *hsa-miR-423*, *hsa-miR-139*, *hsa-miR-141*, and *hsa-miR-29c* in serum exosomes can be protected from RNase degradation and were transferred into target cells and subsequently regulated the expression of certain mRNAs in recipient cells. Specifically, both exosomal *hsa-miR-141* and *hsa-miR-29c* may regulate the expression of *GCNT2* to modulate CRC metastasis. Exosomal *has-miR-423* probably involves in the process of MSI by regulating *SLC39A10* and *FOXQ1*. Moreover, exosomal *hsa-miR-139* could target *SLC39A10*, *KIAA1549*, *FUTI*, and *LZTS1* to exert several biological functions in CRC development. Nevertheless, the validation of these exosomal miRNA-mRNA pairs is required for elucidating their precise mechanism underlying CRC development.

Collectively, the current study provides a picture of the patho-mechanism of CRC and provides avenues for further investigation and screening of efficient biomarkers for therapeutic intervention in CRC. Some limitations of this study should be noted. First, the normal sample size was relatively small, but we applied random sampling and multiple trials to minimize the deviations. Besides, our finding is a preliminary step to reveal a novel exosomal miRNA-mRNA network for CRC; further experimental verification and large cohorts are required to determine clinical usefulness of the five exosomal hub miRNAs and their target mRNAs as biomarkers for CRC. The diagnostic value of the five hub exosomal miRNAs should be verified in large-scale and multi-center studies of CRC patients, and their potential clinical significance should be evaluated by comparing clinical and pathological characteristics in these samples. The exosomal miRNA-mRNA network should be verified by in vitro and in vivo studies.

## Conclusions

Our study efficiently identified several candidate targets (*CBFB*, *CDH3*, *ETV4*, *FOXQ1*, *FUT1*, *GCNT2*, *GRIN2D*, *KIAA1549*, *KRT80*, *LZTS1*, *SLC39A10*, *SPTBN2*, *ZSWIM4*, *hsa-miR-126*, *hsa-miR-139*, *hsa-miR-141*, *hsa-miR-29c*, and *hsa-miR-423*) that can potentially serve as biomarkers in the diagnosis of CRC, and revealed an exosomal miRNA-mRNA network in CRC progression. These findings provide a new direction for diagnosis and treatment of CRC.

## Supplementary Information

**Additional file 1. Table S1**. The differential expressed miRNA/mRNA identified by edgeR and limma.

**Additional file 2. Table S2**. The targeted mRNA of the candidate exosomal miRNAs.

## Data Availability

The datasets used and/or analysed during the current study are available from NCBI Gene Expression Omnibus (GEO: GSE39833) https://www.ncbi.nlm.nih.gov/geo/ and the cancer genome database (TCGA: COAD, READ) https://portal.gdc.cancer.gov.

## Abbreviations

CRC: Colorectal cancer
CEA: Carcinoembryonic antigen
LASSO: Least absolute shrinkage and selection operator
COAD: Colon adenocarcinoma
ROC: Receiver operating characteristic
TCGA: The cancer genome database
READ: Rectal adenocarcinoma
GEO: Gene Expression Omnibus
DEA: Differential expression analysis
BP: Biological process(s)
GO: Gene Ontology
CC: Cellular component
MF: Molecular function
KEGG: Kyoto Encyclopedia of Genes and Genomes
OS: Overall survival
